# Rivaroxaban Versus Apixaban: A Comparison Without a Simple Solution

**DOI:** 10.1016/j.mayocpiqo.2024.05.004

**Published:** 2024-06-11

**Authors:** Marc Cohen, Alex C. Spyropoulos, Shaun G. Goodman, Sarah A. Spinler, Marc P. Bonaca, Theresa M. Redling, Gautam Visveswaran, Sumit Sohal

**Affiliations:** a Department of Medicine, Newark Beth Israel Medical Center, Newark, NJ; b Division of Cardiology, Department of Medicine, Newark Beth Israel Medical Center, Newark, NJ; c Department of Medicine, Rutgers-New Jersey Medical School, Newark, NJ; d Anticoagulation and Clinical Thrombosis Services, Northwell Health at Lenox Hill Hospital, New York, NY; e Department of Medicine, Donald and Barbara Zucker School of Medicine at Hofstra/Northwell, Hempstead, NY; f Institute of Health System Science, The Feinstein Institutes for Medical Research, New York, NY; g Division of Cardiology, Department of Medicine, St. Michael’s Hospital Professor and Heart & Stroke Foundation of Ontario (Polo), Toronto, Ontario, Canada; h Department of Medicine, University of Toronto Consultant, Ontario, Canada; i Canadian Heart Research Centre, Canadian VIGOUR Centre Ontario, Canada; j Department of Pharmacy Practice, Binghamton University School of Pharmacy and Pharmaceutical Sciences, Binghamton University, Binghamton, NY; k Division of Cardiology and Vascular Medicine, Department of Medicine, University of Colorado School of Medicine, Aurora; l Division of Geriatric Health and Disease Management, Cooperman Barnabas Medical Center Maida Geriatric Institute, Livingston, NJ; m Division of Cardiology, Department of Medicine, Yale New Haven Hospital, New Haven, CT

Since the original Beers Criteria were developed in 1991 and subsequently expanded in 1997, the American Geriatric Society (AGS) Beers Criteria has become a very useful source of information to optimize patient safety and minimize patient harm in older adults (age older than 65 years).^1^ Recently, the AGS published its 2023 updated AGS Beers Criteria for potentially inappropriate medication use in older adults.^2^ This 2023 publication reviewed evidence published between 2017 and 2022 in order to update the previously published AGS 2019 Beers Criteria. The review we present below is focused solely on the recommendations within the 2019 and 2023 AGS publications pertaining to anticoagulation with the direct acting oral anticoagulants (DOACs).

We are concerned with the changes in the AGS recommendations for rivaroxaban, relative to apixaban, outlined in the 2023 publication.^2^ As stated in the 2023 publication, “The recommendation for rivaroxaban has changed from ‘use with caution,’ to ‘avoid’ for long-term treatment of nonvalvular atrial fibrillation (NVAF) and venous thromboembolism (VTE), with the rationale being that observational studies and network meta-analyses find that this drug confers a higher risk of major and gastrointestinal (GI) bleeding in older adults than other DOACs, particularly apixaban but also dabigatran.”^2^ The basis of this text is concerning to us as health care providers for older adults (age older than 65 years), who require oral anticoagulation.

### Methods

We originally reviewed the original peer-reviewed, randomized controlled trials’ (RCTs) data collected from the rivaroxaban and apixaban registration trials,^3^, ^4^, ^5^, ^6^, ^7^ specifically regarding the subgroup of older adults. The original Rivaroxaban Once Daily Oral Direct Factor Xa Inhibition Compared with Vitamin K Antagonism for Prevention of Stroke and Embolism Trial in Atrial Fibrillation (ROCKET AF trial)^3^^,^^4^ analyses had already identified an increased rate of GI bleeding rate relative to warfarin, which is included in the US Food and Drug Administration (FDA) approved label. Subsequently, we conducted a literature search using the search term, *direct acting oral anticoagulants*, in PubMed from June 1, 2017, to May 31, 2023, and screened all full-text English studies comparing differences in outcomes within the class of direct acting oral anticoagulants. We included RCTs, cohort studies and meta-analysis. We excluded duplicated articles, review, case reports, case series, and studies that did not provide direct comparison of DOACs. Full texts of all eligible studies were retrieved. A total of 339 studies were initially identified of which 230 studies were excluded based on the exclusion criteria. On review of the remaining 109 studies, full texts were reviewed, and 41 studies were included for final review^8^, ^9^, ^10^, ^11^, ^12^, ^13^, ^14^, ^15^, ^16^, ^17^, ^18^, ^19^, ^20^, ^21^, ^22^, ^23^, ^24^ (Figure 1). Not all studies reviewed are referenced further. No randomized studies were conducted comparing the direct oral anticoagulants with each other during this time frame. All studies were assessed for their quality of evidence using the American College of Physicians–based approach^25^ and GRADE–based approach.^26^ On the basis of either approach, all observational studies were of “low” quality, whereas all RCTs were of “high-quality” and with “low bias.”

**Figure 1:**
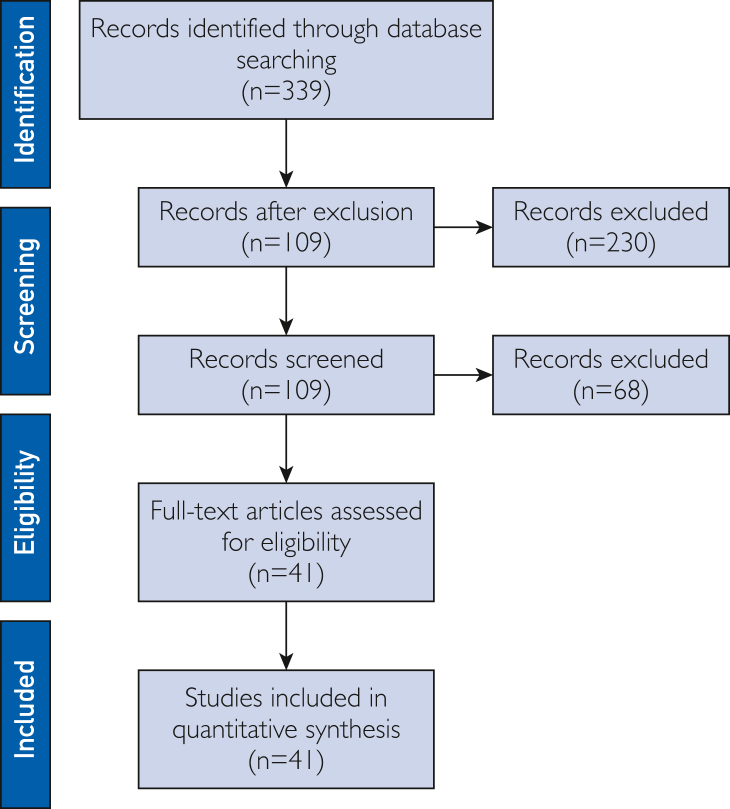
PRISMA flow diagram. PRISMA, Preferred Reporting Items for Systematic Reviews and Meta-Analyses.

### Results

1. The AGS is making a specific therapeutic recommendation, regarding 2 very effective drugs that are US FDA approved for use in all adult age groups. The 2023 AGS Beers Criteria are relying on “observational studies and network meta-analyses.”^2^

Several elements regarding the data derived from the original, prospective, randomized DOAC trials, include, the median age of patients enrolled in ROCKET AF trial was 73 years.^3^^,^^4^ Twenty-five percentage of more than 14,000 randomized ROCKET AF trial patients were 78 years or older, and the follow-up was greater than 1.5 years. The median age of the 18,000 patients enrolled in Apixaban for Reduction in Stroke and Other Thromboembolic Events in Atrial Fibrillation (ARISTOTLE),^7^ the apixaban registration trial, was 70 years, and they were followed up for more than 1.5 years. The 2023 AGS recommendations are made in the absence of any direct, head-to-head rivaroxaban vs apixaban prospective, randomized trials in older adults incorporating blinded analysis of clinical outcomes. Therefore, there was no high-quality evidence^2^—for the change in AGS recommendation.

2. The AGS recommendation indicates to “avoid [rivaroxaban] for long-term treatment of nonvalvular atrial fibrillation and VTE.”

The original randomized ROCKET AF trial^3^^,^^4^ and ARISTOTLE^7^ trial had median follow-ups of 1.6 and 1.8 years, respectively. In many of the recent observational studies we reviewed,^8^, ^9^, ^10^, ^11^, ^12^, ^13^, ^14^, ^15^, ^16^, ^17^, ^18^, ^19^, ^20^, ^21^, ^22^, ^23^, ^24^ many had follow-up durations of only 3 to 6 months. All major outcomes in ROCKET AF trial^3^^,^^4^ and ARISTOTLE^7^ vs vitamin K antagonists were adjudicated by committee members who were blinded to treatment allocation.

A major weakness of shorter-term follow-up of patients with NVAF and/or deep vein thrombosis (DVT)/pulmonary embolism treated with anticoagulant therapies is the potential difference in the event rates over time relating to individual end points. Major bleeding (MB) end points are often early, that is, “frontloaded,” and demonstrate some flattening/tapering over longer-term follow-up.^27^ In contrast, the risk of stroke and/or systemic embolism (SSE) appears to be a relatively stable, constant function over time (Figure 2).^27^ Therefore, short-term studies may have overrepresentation of MB rates and hazard ratios compared with thrombotic end points such as SSE or DVT/VTE.

**Figure 2:**
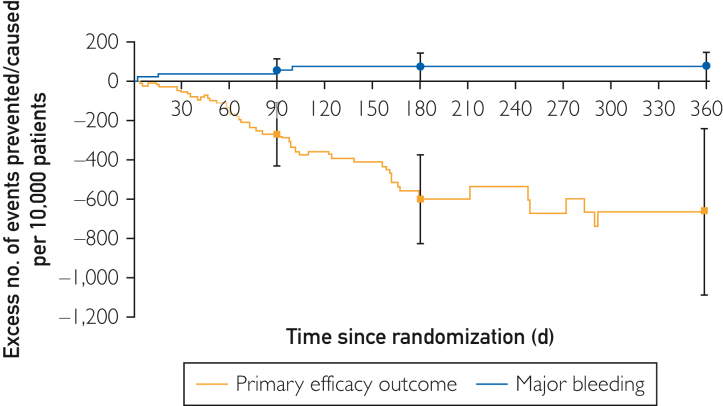
Kaplan-Meier rate differences per 10,000 patients for symptomatic VTE and major bleeding until the end of study treatment. Reproduced with permission from Wells et al.^27^ VTE, venous thromboembolism.

Among the observational trials that we reviewed, we identified 15 studies that had an average follow-up of less than 180 days. Eighteen studies had an average follow-up approaching 1 year. Only 8 studies had an average follow-up of more than 1 year. This broad range of follow-up among the observational studies has potential to introduce time bias on consistency of the measured end points.

Anticoagulants for Reduction in Stroke: Observational Pooled Analysis on Health Outcomes and Experience of Patients (ARISTOPHANES) Elderly study,^18^ a very large claims-based study (n=37,000) suggested a significant (*P*<0.05), favorable profile for apixaban vs rivaroxaban. This study was a subgroup analysis derived from the ARISTOPHANES study^8^ and focused only on NVAF patients aged 80 years or older. The patients were newly treated with a DOAC as per the pharmacy claims between 2013 and 2015. Although the strength of this analysis was the large volume of patient data, the mean follow-up was substantially less than 1 year. Follow-up for the apixaban vs the rivaroxaban patients was only 210 and 248 days, respectively. Unfortunately, the median follow-up time among the matched cohorts was only 5 to 6 months. Using the metric of absolute rate difference per 100 patient-years (ARD/100 PY), this study^18^ suggested a significant (*P*<0.05), favorable profile for apixaban vs rivaroxaban. Compared with apixaban, rivaroxaban had an excess SSE of 0.53/100 PY, an excess MB of ARD/100 PY of 4.24, and an excess GI bleed of ARD/100PY of 2.64.

Another real world, active-comparator, retrospective cohort study, was published by Fralick et al^15^ in 2020 focusing on patients with NVAF. This study used a nationwide commercial health care claims database from 2012 to 2019 in which 39,351 newly prescribed apixaban patients were propensity score matched with 39,351 patients with newly prescribed rivaroxaban. Their mean age was 69 years, and in contrast to ARISTOPHANES Elderly, the mean follow-up was 288 days for apixaban users and 291 days for rivaroxaban users. In addition, this study excluded patients using lower dosages of either medication (rivaroxaban <20 mg daily or apixaban < 5 mg twice daily). All patients were followed up for up to 365 days, unless they reached the end of the study period, dis-enrolled, experienced a study outcome, or died. The authors noted the confounding patient behavior of treatment discontinuation and switching to the comparator medication. Rivaroxaban had an excess SSE compared with apixaban of only 0.14/100 PY, an excess MB of ARD/100 PY of 1.2, and an almost identical rate of intracranial hemorrhage (ICH) to apixaban. In contrast to ARISTOPHANES Elderly, this study suggested a much less dramatic, if any, difference of clinical outcomes favoring apixaban over rivaroxaban.

A large retrospective cohort study of patients with cancer-associated VTE and low risk of bleeding (OSCAR-US) compared 1093 patients on rivaroxaban and 1344 patients on apixaban with the outcome of developing recurrent VTE or any bleed resulting in hospitalization at 3 and 6 months.^28^ The authors used inverse probability of treatment-weighted Cox regression to calculate hazard ratios (HRs) and 95% CIs. Rivaroxaban was found to have similar hazard to apixaban for the composite outcome at 3 months (HR, 0.87; 95% CI, 0.60-1.27) and 6 months (HR, 1.00; 95% CI, 0.71-1.40) and for any other outcome at 3 or 6 months.^28^

A recent meta-analysis of observational studies assessing 24,156 patients compared rivaroxaban with apixaban in the treatment of VTE using both fixed-effects and random effects modeling.^29^ Although the authors found no differences in VTE recurrence with apixaban (RR, 0.77; 95% CI, 0.57-1.04), there was a significant reduction in MB favoring apixaban (RR, 0.68; 95% CI, 0.61-0.76). However, the studies had short durations of follow-up of only 3 to 6 months.^29^

The study by Talmor-Barkan et al^12^ published in 2022 derived data from patients with NVAF enrolled in the Israeli health care organization CLALIT, with follow-up started at first eligible DOAC prescription and ended at an outcome event including death, treatment discontinuation, disenrollment from the health care organization, end of follow-up (6 years after index event), or the end of the study (May 1, 2020), whichever occurred first. This analysis included 56,553 patients on different DOACs among which there were 35,101 on apixaban and 15,682 on rivaroxaban. In contrast to the ARISTOPHANES Elderly and the study by Fralick et al^15^, mortality and ischemic stroke rates were lower with rivaroxaban vs apixaban (HR, 0.88 and 0.92, respectively; *P*<0.04). No significant differences in the rates of myocardial infarction, SSE, and overall bleeding were noticed between the different DOACs groups. Using the metric of ARD/100 PY, this study suggested a more favorable profile for rivaroxaban vs apixaban. Compared with rivaroxaban, apixaban had an excess ischemic stroke rate of 0.7/100 PY, whereas rivaroxaban had an excess MB ARD/100 PY of 0.98, and a GI bleed ARD/100 PY of 0.16. The authors concluded, “We found significant differences in outcomes between the 3 studied DOACs. The results emphasize the need for RCTs that will compare rivaroxaban, apixaban, and dabigatran in order to better guide the selection among them.”^12^ The 6-year follow-up allowed for generation of Kaplan-Meier survival curves of all-cause mortality (Figure 3),^12^ suggesting rivaroxaban had an equal or slightly better all-cause mortality rate than apixaban regardless of the increased GI bleed rate seen with rivaroxaban. In addition, Talmor-Barkan et al^12^ identified that in the real world, greater than 24% of patients with NVAF treated with DOACs were using off-label doses, with most being underdosed. They summarized their results as follows: “The long follow-up data of 6 years may reveal differences in mortality risk in favor of rivaroxaban that were not found in previous studies in which the follow-up period was shorter. We found that the differences in mortality and ischemic stroke are age-related. A comparison between apixaban and rivaroxaban revealed decreased GI bleeding in the apixaban group and decreased ICH in the rivaroxaban group. We believe that the present study emphasizes the need for future RCTs that will compare apixaban, rivaroxaban and dabigatran in order to better guide the use of the different DOACs in clinical practice.”^12^ As an example of how large a randomized trial is needed, in the setting of VTE to design a future RCT with a head-to-head comparison of 2 DOACs with 90% power and a 2-sided α of 0.05 would require approximately 45,000 patients, given low rates of both recurrent VTE and MB in the DOAC VTE registration trials.

3. The AGS’ 2023 therapeutic recommendation was based on, “with the rationale being that observational studies and network meta-analyses find that this drug [rivaroxaban] confers a higher risk of major and gastrointestinal bleeding in older adults…”

**Figure 3:**
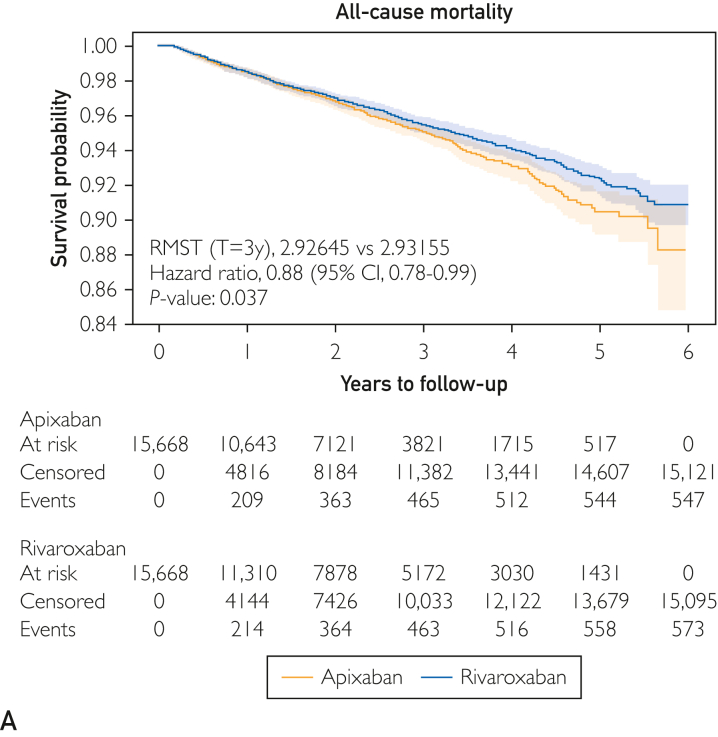
A propensity score–matched Kaplan-Meier curve for all-cause mortality in patients with atrial fibrillation treated with apixaban versus rivaroxaban in a 6-year follow-up. Reproduced with permission from Talmor-Barkan et al.^12^ RMST, restricted mean survival time.

It is of concern that the basis of any therapeutic decision be dependent solely on a safety end point GI bleeding, without focusing on the balance between primary efficacy relative to the safety outcomes, that is, “net clinical benefit.” A perspective published by the US FDA entitled, “Weighing Benefits and Risks – The US FDA’s Review of Prasugrel”^30^ highlighted the need for balance stating “the components of the primary end point represented irreversible tissue damage and concluded that the benefits of preventing such events is generally worth the risk of bleeding events that have no irreversible consequences.” In references to older adults, the US FDA further stated, “However, older patients… particularly high risk patients… The US FDA made sure that prasugrel’s label clearly articulates the balance between efficacy and risk – a balance that physicians will need to assess carefully when choosing treatment for individual patients.”^28^ Adverse effects need to be highlighted and their frequency minimized, but in the end, it is the balance that should dictate therapeutic decision making. Two published reports quantified benefit vs risk in NVAF^29^ and long-term DVT/VTE prophylaxis.^27^

Barnett et al^31^ analyzed the prospective, blinded, randomized ROCKET AF trial^3^ data and identified the rate differences for irreversible and reversible outcomes per 10,000 patient-years between rivaroxaban and warfarin. Regarding irreversible events (death, myocardial infarction, SSE, fatal, and/or critical organ bleeding), rivaroxaban had an advantage over warfarin with an ARD/100 PY of 1.3/100 PY.^31^ Regarding nonfatal or noncritical organ major bleeding, rivaroxaban had an excess in MB of 0.56 ARD/100 PY. The excess in MB identified in ROCKET AF trial is clearly identified in the package insert for Xarelto. In a post hoc net clinical benefit analysis from the ROCKET AF trial,^3^ dichotomized by age older vs younger than 75 years, Halperin et al^4^ focused on the irreversible events of all-cause mortality, life-threatening bleeding, and nonfatal nonhemorrhagic stroke. Analysis of the balance between irreversible events and nonfatal bleeding events suggested that rivaroxaban was more favorable than warfarin in older persons compared with those younger than 75 years.

By contrast, most observational studies published between 2017 and 2023 did not clearly delineate benefit vs risk analyses. A similar benefit-risk analysis was performed on the prospective, randomized EINSTEIN Extension trial patient data set^27^ whose patients were followed up for a minimum of 1 year. They compared the absolute rate differences among the irreversible (primary efficacy outcome), as well as the nonfatal reversible end point of MB. Their analysis suggested that for patients with DVT/VTE, extending their prophylaxis with rivaroxaban vs placebo had a significantly favorable net clinical benefit. This rate difference was even more significant in the older subgroup aged older than 75 years compared with younger patients. Another very important lesson emerged from their comparison of efficacy vs bleeding events that could only be appreciated as a consequence of their longer follow-up of at least 1 or more years (Figure 2). The figure shows that the time activity for MB was frontloaded in the first 3-4 months and subsequently was flat. By contrast, recurrent DVT/VTE appears to have an ongoing residual risk over time, at least over 6 months. Wells et al^27^ concluded, “The reduction in recurrent VTE with rivaroxaban started early and continued to improve throughout the course of treatment. The increase in MB developed gradually and plateaued at approximately 100 days, suggesting that throughout the course of treatment, benefit would exceed risk.”

By contrast, a 2019 observational study by Dawwas et al^9^ analyzed the retrospective claims made data of over 36,000 patients with DVT/VTE comparing rivaroxaban with apixaban. Their ARD/100 PY for GI bleeding was 3.6, suggesting that apixaban was safer than rivaroxaban. However, their analysis reflected a median of only 102-105 days of follow-up. As stated earlier, short-term studies will be more likely to capture the early event of MB, but that only studies with longer-term follow-up will show a more valid benefit-risk analysis over time.

4. Quality of evidence: The 2023 AGS Beers Criteria are relying on “observational studies and network meta-analyses,” which the AGS elsewhere describes as “moderate-quality” or “low-quality.”^2^

Despite the lack of high-quality randomized trial data, AGS^2^ states that its 2023 recommendations are of moderate-quality of evidence, with strength of recommendation being strong. According to the AGS, “Strength of recommendation ratings for each criterion are based on synthetic integration of the quality of evidence, the frequency and severity of potential adverse events and their relationship to potential benefits, and clinical judgment. ‘Strong’; Harms, adverse events, and risks clearly outweigh the benefits. ‘Weak’; Harms, adverse events, and risks may not outweigh the benefits.”^2^ The consensus that “risks clearly outweigh the benefits,” based on moderate or low-quality, retrospective, nonrandomized studies, many of which had short duration, is in our opinion not supportable. Another point to consider is the fact that the relative/absolute differences between DOACs see in the observational trial cited, often exceed those differences observed in the prospective blinded DOAC vs vitamin K antagonist randomized trials. This is counterintuitive and suggests that the observational studies and network analyses, without any direct DOAC comparison arms, are confounded.

### Conclusion

It is our belief, that the 2019 recommendation “use with caution” for rivaroxaban is supported by high-quality prospective randomized trials and post hoc analyses of randomized data.^3^, ^4^, ^5^, ^6^ These trials with blinded clinical events committees found a clear-cut benefit regarding irreversible stroke and ICH but with a concomitant increase in nonfatal MB primarily accounted for by GI bleeding relative to vitamin K antagonists. However, the more recent 2023 AGS Beers Criteria recommendation,^2^ “avoid for long-term use,” prompted us to review the many reports published between 2017 and 2023. It is our belief that the 2023 recommendation to avoid rivaroxaban in favor of apixaban for long-term use is not adequately substantiated by the nonrandomized studies. Many of these studies have very short follow-up periods, and many do not clearly articulate the balance between efficacy and risk. Further, many of these studies cannot account for the confounding role of frequent prescribing of inappropriate reduced dosing of DOACS.^32^

In contrast to the 2023 AGS recommendations, multiple other Society guidelines recommend the use of rivaroxaban for NVAF and/or VTE. They include but are not limited to the American Heart Association, American College of Cardiology, American Stroke Association, European Society of Cardiology, 2020 Canadian Cardiovascular Society, Heart Rhythm Society,·American Academy of Family Physicians, American Academy of Neurology, American College of Chest Physicians, and the American Society of Hematology.

We are now in the age of large data and “population health.” We are also susceptible to the mindset of “this study will never be done; no one will sponsor it.” Our belief is that in the absence of high-quality randomized trials, it is important to appreciate the general trends observed in carefully analyzed observational studies. However, we cannot and should not default to observational studies to set therapeutic decisions. Rather, we should insist on testing any serious outcome conclusions seen in the observational studies by undertaking high-quality prospective, randomized trials before changing clinical practice. Multicenter collaboration led to the very successful Global Utilization of Streptokinase and Tissue Plasminogen Activator for Occluded Coronary Arteries and Thrombolysis In Myocardial Infarction trials of multiples of 100,000 patients. Despite frustration that a study cannot be done or would not be supported, multiple prospective randomized studies were properly executed during the worse infectious pandemic of our time, coronavirus disease.^33^^,^^34^

## Potential Competing Interests

Dr Cohen reports speakers bureau fees from 10.13039/100008897Janssen Pharmaceuticals. Dr Spyropoulos reports grant/research payments from 10.13039/100008897Janssen Pharmaceuticals, 10.13039/100004325AstraZeneca and Boehringer Ingelheim, receives consulting fees from 10.13039/100005565Janssen, 10.13039/100004325AstraZeneca, Bristol Meyer Squibb, 10.13039/100004339Sanofi, 10.13039/100004326Bayer, and 10.13039/100004319Pfizer. Dr Bonaca is the Executive Director of CPC, a non-profit academic research organization affiliated with the University of Colorado, that receives or has received research grant/consulting funding between February 2021 and present from 10.13039/100001316Abbott Laboratories, Adamis Pharmaceuticals Corporation, Agios Pharmaceuticals, Inc., Alexion Pharma, Alnylam Pharmaceuticals, Inc., Amgen Inc., Angionetics, Inc., ARCA Biopharma, Inc., Array BioPharma, Inc., AstraZeneca and Affiliates, Atentiv LLC, Audentes Therapeutics, Inc., Bayer and Affiliates, Beth Israel Deaconess Medical Center, Better Therapeutics, Inc., BIDMC, Boston Clinical Research Institute, Bristol-Meyers Squibb Company, Cambrian Biopharma, Inc., Cardiol Therapeutics Inc., CellResearch Corp., Cook Medical Abbott Laboratories, Adamis Pharmaceuticals Corporation, Agios Pharmaceuticals, Inc., Alexion Pharma, Alnylam Pharmaceuticals, Inc., Amgen Inc., Angionetics, Inc., ARCA Biopharma, Inc., Array BioPharma, Inc., AstraZeneca and Affiliates, Atentiv LLC, Audentes Therapeutics, Inc., Bayer and Affiliates, Beth Israel Deaconess Medical Center, Better Therapeutics, Inc., BIDMC, Boston Clinical Research Institute, Bristol-Meyers Squibb Company, Cambrian Biopharma, Inc., Cardiol Therapeutics Inc., CellResearch Corp., Cook Medical Incorporated, Covance, CSL Behring LLC, Eidos Therapeutics, Inc., EP Trading Co. Ltd., EPG Communication Holdings Ltd., Epizon Pharma, Inc., Esperion Therapeutics, Inc., Everly Well, Inc., Exicon Consulting Pvt. Ltd., Faraday Pharmaceuticals, Inc., Foresee Pharmaceuticals Co. Ltd., Fortress Biotech, Inc., HDL Therapeutics Inc., HeartFlow Inc., Hummingbird Bioscience, Insmed Inc., Ionis Pharmaceuticals, IQVIA Inc., JanOne Biotech Holdings Inc., Janssen and Affiliates, Kaneka, Kowa Research Institute, Inc., Kyushu University, Lexicon Pharmaceuticals, Inc., LSG Kyushu University, Medimmune Ltd., Medpace, Merck & Affiliates, Novartis Pharmaceuticals Corp., Novate Medical, Ltd., Novo Nordisk, Inc., Pan Industry Group, Pfizer Inc., PhaseBio Pharmaceuticals, Inc., PPD Development, LP, Prairie Education and Research Cooperative, Prothena Biosciences Limited, Regeneron Pharmaceuticals, Inc., Regio Biosciences, Inc., Rexgenero, Sanifit Therapeutics S.A., Sanofi-Aventis Groupe, Silence Therapeutics PLC, Smith & Nephew plc, Stealth BioTherapeutics Inc., State of Colorado CCPD Grant, The Brigham & Women's Hospital, Inc., The Feinstein Institutes for Medical Research, Thrombosis Research Institute, University of Colorado, University of Pittsburgh, VarmX, Virta Health Corporation, WCT Atlas, Worldwide Clinical Trials Inc., WraSer, LLC, and Yale Cardiovascular Research Group, receives support from the AHA SFRN under award numbers 18SFRN3390085 (BWH-DH SFRN Center) and 18SFRN33960262 (BWH-DH Clinical Project), received consulting fees from Audentes and reports modest stock holdings in Medtronic and Pfizer. The other authors report no competing interests.
